# Diagnostic dilemmas in abdominal wall tumors: surgical implications and use of mesh for primary repair: case series

**DOI:** 10.1093/jscr/rjag414

**Published:** 2026-05-31

**Authors:** Diana Lilian Torres Dávila, Carlos Vicente Mejía Ochoa, María Silvana Montúfar Flores, Gabriela Alejandra Orbe Reyes, Erika Deyanira Montenegro Garcia, David Ignacio Narváez Salas, Fernando Israel Zumárraga López, Christian Paul Jara Santamaria

**Affiliations:** Department of General Surgery, Hospital de Especialidades Eugenio Espejo, Av. Gran Colombia s/n y Yaguachi, 170403 Quito, Ecuador; Department of Oncologic Surgery, Hospital de Especialidades Eugenio Espejo, Av. Gran Colombia s/n y Yaguachi, 170403 Quito, Ecuador; Department of General Surgery, Hospital de Especialidades Eugenio Espejo, Av. Gran Colombia s/n y Yaguachi, 170403 Quito, Ecuador; Department of General Surgery, Clinica Occidental de Especialidades Neovida, OE9A N47-56 y Francisco Montalvo, 170528 Quito, Ecuador; Pontificia Universidad Católica del Ecuador, Department of General Surgery, Hospital Padre Carollo, Av. Rumichaca S33-10, 170702 Quito, Ecuador; Department of General Surgery, Hospital Vozandes Quito, Av. Juan José de Villalengua Oe2-37, 170521 Quito, Ecuador; Pontificia Universidad Católica del Ecuador, Department of General Surgery, Hospital de Especialidades Carlos Andrade Marín, Av. Ayacucho N19-63, 170103 Quito, Ecuador; General Surgeon Hospital Axxis, Av. 10 de Agosto N39-155 y Diguja, 170104 Quito, Ecuador

**Keywords:** desmoid tumor, adenocarcinoma, endometrioma, abdominal wall surgery, metastasis

## Abstract

Abdominal wall tumors represent a significant challenge in surgical practice due to their wide range of etiologies and nonspecific clinical presentation. These lesions can include benign, malignant, and pseudotumoral pathologies, such as desmoid tumors, abdominal wall endometriomas, and adenocarcinoma metastases. Inadequate evaluation can lead to unnecessary aggressive treatments or the omission of oncologic pathology. This study presents a series of three cases with the aim of establishing more precise diagnostic and therapeutic strategies.

## Introduction

Abdominal wall surgery faces significant diagnostic and therapeutic challenges due to the presence of desmoid tumors, endometriomas, and adenocarcinoma metastases, where their incidence is extremely rare. Desmoid tumors, also known as aggressive fibromatosis, are rare neoplasms that constitute ~0.03% of all tumors and 3% of soft tissue tumors, with an estimated incidence of 2 to 4 cases per million people per year [1]. On the other hand, endometriomas in the abdominal wall, a form of extrapelvic endometriosis, occur in 0.03%–1.5% of women after a cesarean section or pelvic surgery [2]. In the case of abdominal wall metastases, these are uncommon, but can occur in 0.7%–9% of cases, especially in patients with a history of gastrointestinal or gynecological cancer [3]. These findings underscore the importance of a thorough clinical and imaging evaluation in surgical practice for accurate diagnosis and proper management of these conditions.

Here, we report three cases of abdominal wall tumors which have been a diagnostic and therapeutic challenge in abdominal wall surgeries.

## Case reports

### Case 1

This is a 21-year-old female patient with no significant medical or surgical history. She presented with a progressive growth of a mass in the abdominal wall, accompanied by pain in the tumor, weight loss, and diaphoresis. She attended a secondary care hospital, where a simple computed tomography scan of the abdomen and pelvis was performed, revealing that the tumor was dependent on the rectus abdominis muscles. Physical examination revealed an indurated mass in the abdominal wall measuring ~8 × 11 cm, painful to palpation, with no trophic changes in the skin and no signs of active infection. A biopsy was performed, resulting in a histopathological diagnosis of mesenchymal neoplasia with a spindle cell pattern in favor of Desmoid Fibromatosis. Surgical resolution was planned, with an exploratory laparotomy and tumor resection of the right abdominal wall with polypropylene mesh placement and Jackson Pratt drains. Among the findings, a tumor measuring ~20 × 20 cm was evident, dependent on the right rectus abdominis muscle in its entire extension with loss of muscular plane in the posterior leaf and peritoneum. This tumor extended to the internal oblique muscle and ipsilateral transverse abdominis muscle. For abdominal wall plasty, a 30 × 30 cm polypropylene mesh was used in the preperitoneal space with polyglactin 910 3-0 fixation stitches. The patient during the postoperative period, with favorable evolution, tolerating a general diet and with regular pain management, was discharged on the fifth postoperative day without complications. Histopathology of the surgical specimen indicates desmoid-type fibromatosis infiltrating skeletal muscle tissue and surrounding fibroadipose tissue. The patient was monitored for 6 months postoperatively. During this period, there was no clinical or imaging evidence of tumoral recurrence or incisional hernia. Additionally, no complications related to the use of the synthetic mesh, such as chronic pain or infection, were identified.

### Case 2

**Figure 1:**
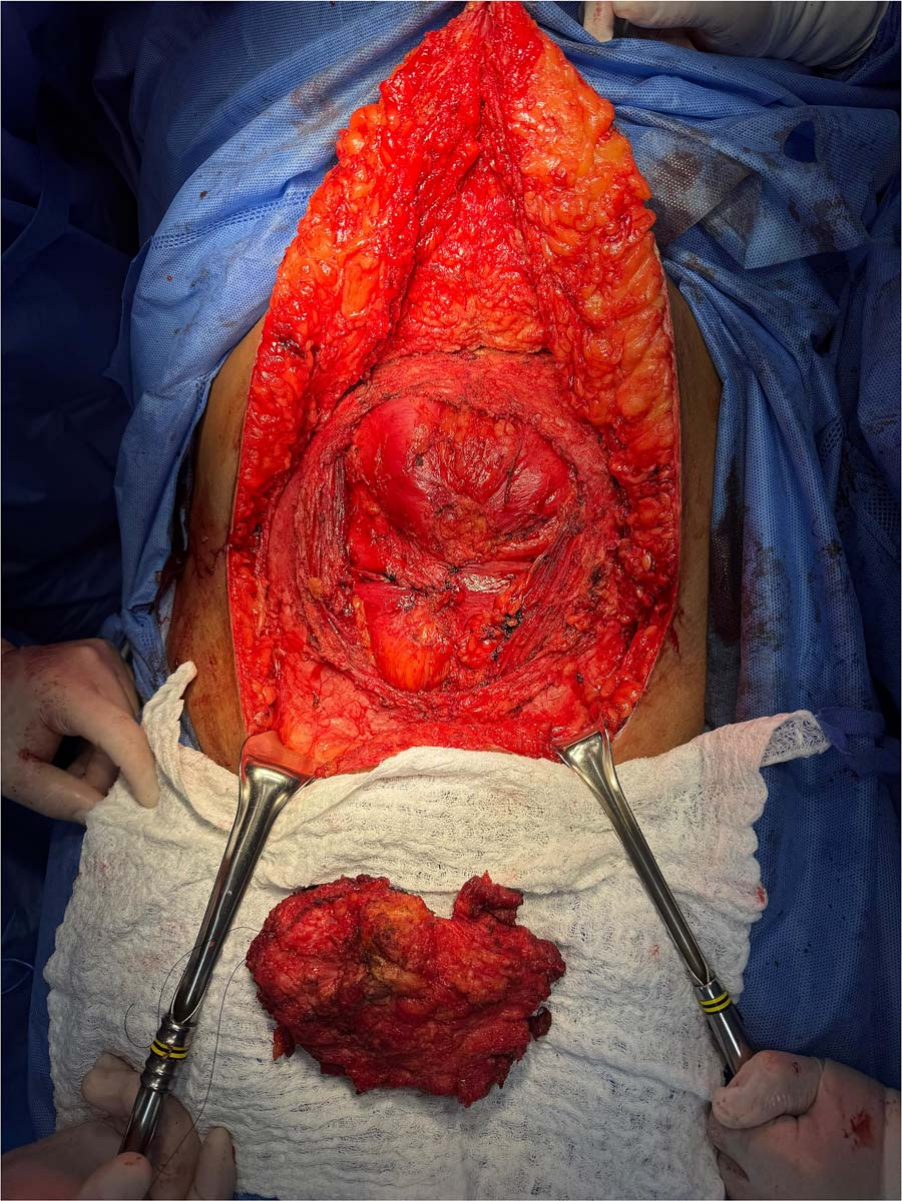
Bloc resection of the abdominal wall infiltrated by endometrioma.

**Figure 2:**
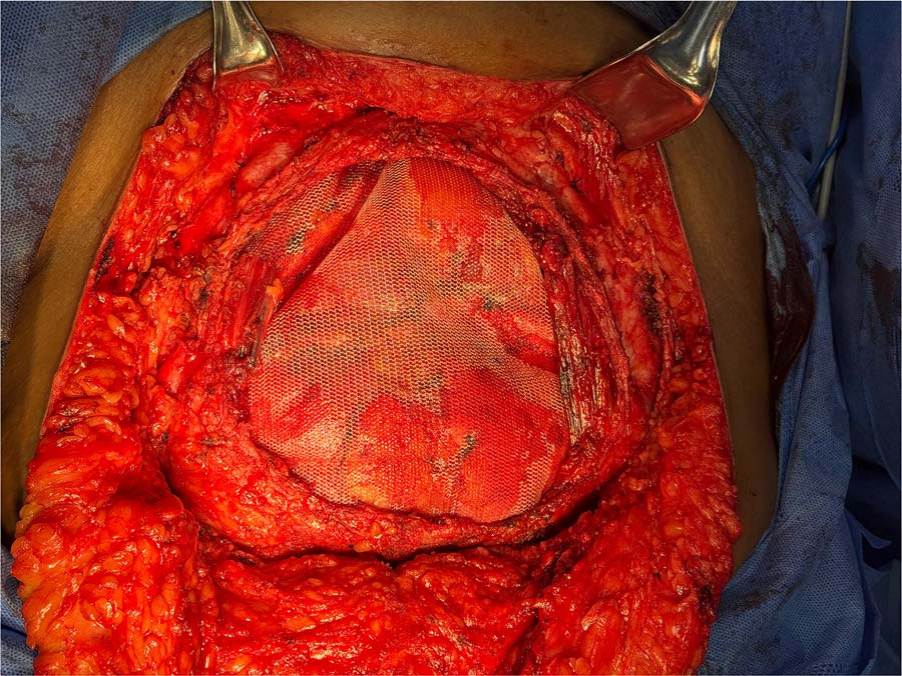
Abdominal wall reconstruction using a polypropylene mesh prosthesis.

**Figure 3:**
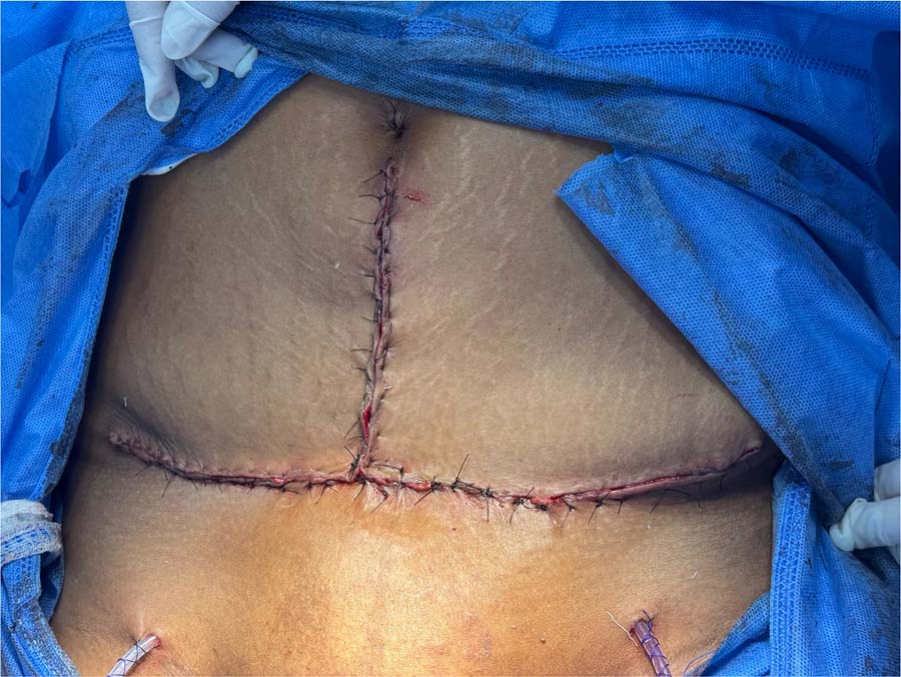
Final intraoperative view of the surgical wound with placement of supra-aponeurotic Jackson-Pratt drains.

A 34-year-old female patient with a clinical history of grade III obesity, high blood pressure, and a surgical history of 4 cesarean sections, salpingectomy, laparoscopic cholecystectomy, and surgery for an abdominal wall mass 4 years ago in a second-level hospital on the Ecuadorian coast. She came to the clinic with a progressively growing mass in recent years, accompanied by tenderness and bleeding through the umbilical scar, which coincides with menstrual cycles. Physical examination revealed the presence of a large fatty panniculus with hypertrophic scars related to previous surgical procedures, and a 10 × 12 cm, painful tumor in the infraumbilical region. A simple and contrast-enhanced tomography of the abdomen and pelvis revealed surgical stigmata in the infraumbilical abdominal wall. The presence of solid lesions with spiculated contours was associated with the 30 mm surgical scar. This was associated with other similar lesions measuring 25 and 15 mm in the subcutaneous tissue attached to the anterior wall of the muscles and inside the lower rectus abdominis muscle. This lesion showed mild post-contrast enhancement. A biopsy of the lesions was performed, revealing tissue compatible with early secretory endometrium. Surgical resolution was scheduled, and resection of the abdominal wall endometrioma was performed (Fig. 1), followed by abdominal wall reconstruction with polypropylene mesh and Jackson Pratt drains (Figs 2 and 3). The findings included a 15 × 10 cm tumor compatible with endometrioma, involving the aponeurosis of the rectus muscles and subcutaneous tissue. A 20 × 20 cm polypropylene mesh was placed retromuscularly, behind the oblique muscles on the left side and the transverse abdominis muscles on the right side. The mesh was secured with prolene and polyglactin 910 1-0. During the postoperative period, the patient developed a superficial infection at the surgical site, which was treated with an ampicillin/sulbactam-based antibiotic regimen. She was discharged on the fifth postoperative day with favorable clinical progress. Two months postoperatively, where the final histopathology result was consistent with endometriosis in deep cuts and free margins. Follow-up assessments were conducted every 3 months for 1 year. No tumoral recurrence or hernia development was observed. Despite an initial superficial surgical site infection treated with antibiotics during the immediate postoperative period, no long-term mesh-related complications occurred.

### Case 3

**Figure 4:**
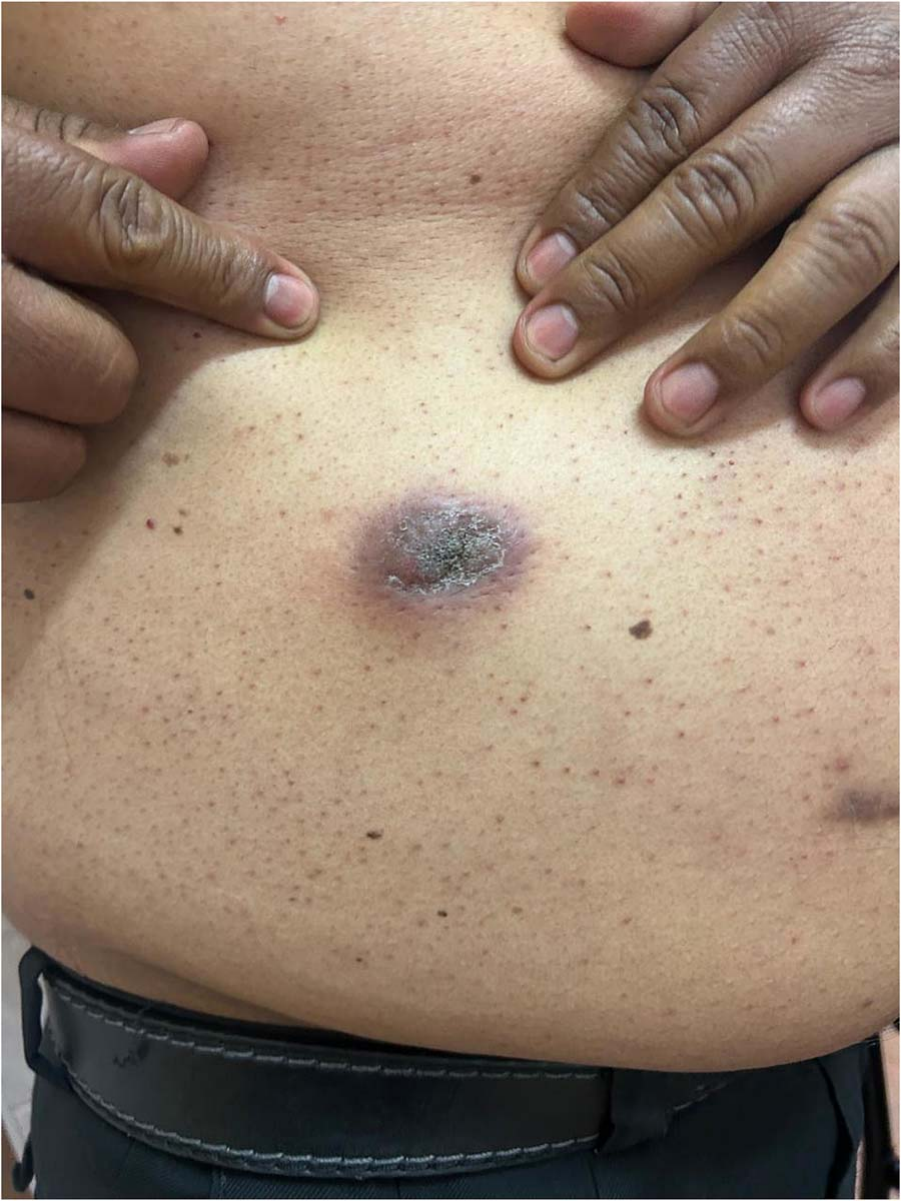
Gradual development of an expanding mass at the right flank trocar insertion site, clinically suggestive of port-site tumor implantation.

A 57-year-old male patient with no significant clinical history and a history of robotic-assisted transversectomy 2 years prior for a diagnosis of well-differentiated adenocarcinoma of the colon, for which he received adjuvant therapy. The patient presented with a progressively growing mass in the surgical wound of the right flank port 6 months prior (Fig. 4). This tumor was painful to palpation and caused intense itching, leading to suspicion of tumor recurrence. Physical examination revealed a soft, depressible abdomen with pain on palpation in the surgical port, where a 3 × 3 cm tumor was evident, with trophic skin changes. To admission for surgical resolution, a colonoscopy was performed, where a high-grade tubular adenoma was evident 90 cm from the anal margin. Prior to surgical intervention, the patient’s case was evaluated by a multidisciplinary team comprising the General Surgery and Surgical Oncology departments. This collaborative review validated the placement of a retromuscular mesh, even in the presence of an active oncologic history, by determining that the functional restoration of the abdominal wall provided a benefit that outweighed the potential risks of local recurrence or prosthetic complications. Additionally, the clinical suspicion of port-site metastasis necessitated and block resection as a definitive measure for both therapeutic symptom control and precise histopathological diagnosis. An exploratory laparotomy was performed with abdominal wall reconstruction. The surgical findings revealed an ulcerated tumor measuring 3 × 3 cm at the robotic access surgical port. This tumor extended to the peritoneum, involving all layers of the abdominal wall. The specimen was excised in block, respecting safety margins, and a 10 × 10 cm retromuscular mesh was placed below the external oblique muscle and fixed with polyglactin 910. Skin flaps were also released to close the defect. The patient was discharged with favorable postoperative progress and assessed in the outpatient clinic 1 month later with the result of a histopathological study showing a diagnosis of primary well-differentiated adenocarcinoma of the colon metastasis. Three follow-up evaluations were conducted at 1, 2, and 6 months postoperatively. No tumoral recurrence at the surgical site or hernia formation was identified. The patient reported no chronic pain or issues related to the prosthetic material. Given that the patient had already received adjuvant therapy following his initial colectomy 2 years prior, the emergence of an abdominal wall metastasis suggests either a failure in previous systemic control or a biological recurrence. Consequently, the patient remains under continuous surveillance by the oncology department to monitor disease progression and adjust management as necessary.

## Discussion

In this case series, we analyze the presentation and management of three entities affecting the abdominal wall: desmoid tumors, endometriomas, and metastases from colorectal adenocarcinoma, as well as the feasibility of mesh closure after resection. Desmoid tumors or aggressive fibromatosis are rare fibroblastic neoplasms, and their parietal location is relatively common in young women. Furthermore, there is an established association with familial adenomatous polyposis, where desmoid disease can develop after abdominal surgery [4].

Abdominal wall endometriosis, typically after cesarean section or other uterine interventions, has a variable incidence in recent series, there are reports ranging between 0.03% and 3.5% in patients with previous uterine surgery and usually manifests as a painful mass with a cyclical character related to menstruation, the definitive diagnosis requires histological confirmation [5, 6].

Wall metastases from colorectal cancer are uncommon but clinically relevant. Current literature describes low rates of seeding in drainage tracts or trocar sites, with an incidence of 1%–4%, although these figures have decreased with improved surgical technique, prophylaxis, and perioperative assessment [7, 8].

Abdominal wall reconstruction after tumor resection should be individualized: in clean surgical fields and moderate defects, retromuscular synthetic mesh offers a lower rate of hernia repair and good functional outcomes. In the presence of contamination or risk of infection, biological mesh or vascularized coverage techniques (flaps, omentoplasty) are preferred. Recent publications support the combined use of barriers and composite meshes and the use of flaps or omentum for protection when implanting a prosthesis in critical circumstances [9, 10].

The optimal timing for abdominal wall reconstruction remains a subject of debate, though recent evidence favors a personalized approach based on the oncological stage and the patient’s nutritional status. Current guidelines suggest that in cases of primary or metastatic tumors, reconstruction should ideally be performed during the same surgical act as the tumor resection (immediate reconstruction) to prevent the loss of abdominal domain and facilitate faster recovery. However, in patients requiring neoadjuvant therapy, it is recommended to delay definitive reconstruction until at least 4 to 6 weeks after the completion of systemic treatment to ensure adequate wound healing and minimize the risk of prosthetic infection. Furthermore, latest consensus emphasizes that a multidisciplinary evaluation is critical to ensure that the reconstructive timing does not interfere with the oncological follow-up or the administration of adjuvant therapies [1, 10].

## Conclusions

In conclusion, although desmoid tumors, endometriomas, and parietal metastases present markedly different etiologies and prognoses, they converge on the diagnostic necessity of a comprehensive multidisciplinary approach. This case series underscores that therapeutic success depends on the combination of precise histological confirmation, rigorous oncological evaluation, and the integration of contemporary systemic therapies. Reconstructive planning must be strictly individualized; in our experience, abdominal wall reconstruction using synthetic meshes in retromuscular or preperitoneal spaces proved to be a safe and effective strategy, achieving the restoration of functional integrity with a zero rate of recurrence or long-term prosthetic complications in follow-ups of up to 1 year.

Furthermore, the management of infrequent complications, such as tumor seeding at surgical ports, must be validated by tumor boards to balance the functional benefit of the repair against the biological risks of the underlying disease. The appropriate selection of prosthetic material and the optimal surgical timing preferably immediate reconstruction in the absence of contamination are critical pillars to minimize morbidity and optimize postoperative outcomes in high complexity centers. Ultimately, the standardization of these clinical and imaging follow-up protocols is essential to ensure patient quality of life and the durability of the surgical repair.
